# Effect of *Broussonetia papyrifera L*. silage on blood biochemical parameters, growth performance, meat amino acids and fatty acids compositions in beef cattle

**DOI:** 10.5713/ajas.19.0150

**Published:** 2019-10-21

**Authors:** Hui Tao, Bingwen Si, Wencai Xu, Yan Tu, Qiyu Diao

**Affiliations:** 1 Feed Research Institute, Chinese Academy of Agricultural Sciences, Key Laboratory of Feed Biotechnology of the Ministry of Agriculture, Beijing 10081, China; 2 National Engineering Research Center of Biological Feed, Beijing 10081, China; 3 Jonathan Technology Limited Development Company, Beijing 101314, China

**Keywords:** *Broussonetia papyrifera* Silage, Antioxidant Capacity, Meat Quality, Amino Acids Composition, Fatty Acids Composition, Beef Cattle

## Abstract

**Objective:**

The study was conducted to investigate the effects of *Broussonetia papyrifera L.* (*B. papyrifera*) silage on growth performance, serum biochemical parameters, meat quality, and meat amino acids and fatty acids compositions in beef cattle.

**Methods:**

Sixty-four male Angus beef cattle were assigned to 4 groups with 4 pens in each group and 4 beef cattle in each pen, and fed with the total mixed ration supplemented with 0%, 5%, 10%, or 15% *B. papyrifera* silage for 100 days (control group, 5% group, 10% group and 15% group) separately.

**Results:**

Beef cattle had significantly higher final body weight (BW) in 15% group, higher average daily gain (ADG) and dry matter intake (DMI) in 5% group, 10% group and 15% group, and higher feed conversion ratio (FCR) in 10% group and 15% group. Significantly higher blood superoxide dismutase (SOD) concentration was noted in 15% group, higher blood total antioxidant capacity (TAC) in 10% group and 15% group, lower 8-hydroxydeoxyguanosine (8-OHdG) and malondialdehyde (MDA) in 15% group. Meat had lower pH in 15% group, higher Commission International DeI’Eclairage (CIE) L* in 5% group, 10% group, and 15% group, and lower drip loss in 15% group. Greater concentration of meat polyunsaturated fatty acids (PUFA) was observed in 10% group and 15% group, and docosahexaenoic acid (DHA) in 15% group.

**Conclusion:**

Diet with 15% *B. papyrifera* silage could improve performance and increase final BW, ADG, DMI, and FCR, enhance the antioxidant functions by decreasing blood 8-OHdG and MDA and increasing blood SOD and TAC, improve the meat quality by lowing pH and drip loss and increasing CIE L*, increase the meat PUFA and DHA concentration. Polyphenols and flavonoids might be the main components responsible for the antioxidant activity and anti-biohydrogenation in the *B. papyrifera* silage. And *B. papyrifera* silage could be used as a new feedstuff in beef cattle nutrition.

## INTRODUCTION

Ruminant products have higher saturated fatty acids (SFA) compared with nonruminants, mainly because of hydrogenation of dietary unsaturated fatty acids by ruminal microbes [1]. Feeding strategies have been developed to increase the unsaturated fatty acids of ruminants’ products by increasing the rumen bypass unsaturated fatty acids or decreasing ruminal hydrogenation of dietary unsaturated fatty acids [2]. For ruminants, there are great opportunities to explore the potential of additional forages in their diets. Meanwhile, the supplements of plant antioxidant extracts or raw antioxidant plant materials in animal diets is a common practice to improve animal performance, health and products [3]. *Broussonetia papyrifera L.* (*B. papyrifera*, paper mulberry), a deciduous tree or shrub in the family Moraceae, natively grows in eastern Asia, are mainly used in papermaking [4], feed [5] and medicine [6] industries. There are about 300,000 hectares of *B. papyrifera* in China, widely distributed in basins of Yellow River, Yangtze River, Pearl River and Mingjiang River. It is reported that certain plants fed to ruminants could reduce the ruminal biohydrogenation [7]. It was found that *B. papyrifera* silage could increase the poly-unsaturated fatty acids (PUFA) concentration in the milk [5]. Besides that, oxidative stress could be very dangerous as no clinical symptoms are obvious, but some metabolites and enzymes in antioxidant defense systems such as vitamins, glutathione peroxidase (GSH-Px), superoxide dismutase (SOD), and catalase (CAT) could prevent oxidative damage, and dietary antioxidants play important roles in preventing damage by free radicals in the system [8]. It is reported that mean levels of total antioxidant capacity (TAC) in shrubs and trees are higher than those in grass, concentrates and timothy hay, which might be beneficial for the TAC status of cattle if they are fed high TAC plants [9]. Many plants contain phytochemicals which have potent antioxidant activities, such as polysaccharides, polyphenolic constituents and antioxidant lignans [10,11]. It is reported that there are antioxidant activities in the fruits, stem, bark and wood of *B. papyrifera* [10,11]. We conducted the experiment to investigate the effects of *B. papyrifera* silage on performance, serum biochemical parameters, meat quality, amino acids and fatty acids composition in male Angus beef cattle.

## MATERIALS AND METHODS

The experimental protocol was approved by the Chinese Academy of Agricultural Sciences Animal Ethics Committee, which was performed in accordance with animal welfare practices and procedures followed the Guidelines for Experimental Animal of the Ministry of Science and Technology (2006, Beijing, China).

### Animal and management

#### Silage preparation

As the *B. papyrifera* sapling grew up to about 1.8 m height, the whole foliage was harvested leaving 20 cm of stubble. Then the crops were chopped to 2.5±1.5 cm, they were wrapped in a size of 70 cm height×70 cm id and 75 kg with a silage wrapper, and then fermented without additive for 45 days before feeding.

### Animal management

Sixty-four male Angus beef cattle with initial body weight (BW) at 495±16.9 kg were allocated in 4 groups randomly with 4 pens in each group and 4 individuals in each pen, i.e., 4 replicates in each group and 4 individuals in each replicate, and given *ad libitum* access to feed and water. The cattle in the control group were fed on the original TMR in the feedlot, and the other 3 treatment groups were fed on the new TMR with 5% (5% group), 10% (10% group) or 15% (15% group) of *B. papyrifera* silage preparation, separately. The four different TMRs were adjusted as isonitrogenous and isoenergetic diets (Table 1) and the fatty acids composition of the diets were shown in Table 2. The beef cattle were fed approximately 110% as expected consumption at 7:00 and 15:00 separately, and the weight of residual was recorded before new feeding were delivered. And the delivery amount was adjusted according to the previous day’s consumption. The feeding adaption period was 7 days, and the experiment period was 100 days.

### Sampling and measurements

The dietary samples, including feeding ingredients, TMRs and residual were collected every 2 weeks during the experiment for analyzing the dry matter (DM), crude fat (ether extract), ash, neutral detergent fiber (NDF), and acid detergent fiber (ADF) according to the method reported by van Soest et al [12] and Goering and van Soest [13], respectively. The pH value of *B. papyrifera* silage was 5.04 and the nutrient composition of *B. papyrifera* silage is shown in Table 1.

At the end of the trial, two beef cattle were selected from each pen and had 24 h feed withdrawal before slaughter. And blood samples were collected from jugular vein for serum. Live BW was recorded before the animals were electrically stunned and slaughtered by exsanguination according to the animals ethics committee of CAAS mentioned above. The hot carcass weight of cattle was recorded for calculating the dressing rate. And then the *Longissimus dorsi* muscle was taken on both sides between 12th thoracic and 5th lumbar vertebrae. The right Longissimus dorsi muscle was kept in −4°C for determination of the meat quality after slaughter. Furthermore, the left *Longissimus dorsi* muscle samples were kept in −80°C for analysis of amino acids and fatty acids composition.

#### Color determination

The cut surface of the muscle was placed at ambient temperature for 30 min, and then lightness (CIE L*), redness ( CIE a*) and yellowness (CIE b*) values, were measured according to the CIE color system (Commission International DeI’Eclairage, 1976) using Minolta CR-100 Chroma Meter (Minolta Camera Co. Ltd, Osaka, Japan) equipped with an 8.0 mm aperture and illuminant C. CIE L*, a*, and b* of meat were recorded 3 times at different positions on the measured surface and averaged for statistical analysis. pH was determined at the same site for muscle color, using a portable Model HI9025 meter (Hanna Instruments, Woonsocket, RI, USA) with a combined electrode by insertion into the at 12th vertebrae end of *Longissimus dorsi* muscle and having a two-point calibration (pH 4.01 and 6.99).

#### Cooking loss determination

The muscle samples were cut into cube pieces with 1.5 cm per side and weighed, then cooked in sealed plastic bags in 80°C water baths until the internal temperature of the meat reached 75°C, which was measured by a 5SC-TT-T-30-36 thermoscouple (Omega Engineering Inc., Stamford, CT, USA) fixed in the geometrical center of the sample. Cooked samples were cooled, fluids were wiped and reweighed. The cooking loss was calculated as a percentage of weight loss [14].

#### Drip loss measurement

Approximately 80 to 100 g muscle samples were cut form *Longissimus dorsi* muscle samples and immediately weighed and suspended in an inflated bag without touching the bag, then they were placed in 4°C refrigerators for 24 h, and then reweighed [15].

#### Shearing force

The muscle samples were cut along the muscle fibers as rectangular cross-section slices of 1.0 cm×1.0 cm ×5.0 cm (length). Then the sample was sheared vertically using a TA. XT Plus/50 texture analyzer (Stable Micro Systems, Surrey, UK). A crosshead speed of 200 mm/min and a 5-kN load cell calibrated to read over a range of 0 to 100 N were applied. Six replicates of each sample were measured to calculate the average value.

#### Blood biochemical parameters

Blood metabolites, immune parameters and anti-oxidation biomarkers were determined by a biochemical auto-analyzer (Hitachi automatic biochemical analyzer 7600, Tokyo, Japan) using commercially available kits: total protein (TP), albumin (ALB), globulin (GLB), non-esterified fatty acid (NEFA), blood urea nitrogen (BUN), total cholesterol (TC), triglyceride (TG), glucose (GLU), high density lipoprotein cholesterol (HDL-C), low density lipoprotein (LDL-C), immunoglobulin A (IgA), IgG, IgM, CAT, SOD, GSH-Px, TAC, 8-hydroxydeoxyguanosine (8-OHdG), advanced oxidation protein products (AOPP), and malondialdehyde (MDA) according to the manufacturer’s instructions. The activity of tumor necrosis factorα (TNFα) was measured by a micro plate reader (BioTek ELX800, BioTek, Winooski, VT, USA) with commercially available kits.

#### Amino acids analysis

About 100 g of muscle samples were homogenized and moved to a tub with 10 mL of 6 mol/L HCl, then snap frozen with liquid nitrogen and later vacuumed-dried. After post-column ninhydrin derivatization, they were hydrolyzed in evacuated sealed tubes with 0.1% phenol for 22 h at 110°C. The solution was diluted and filtered by a 0.45-μm filter, and then the amino acids were determined using an automated amino acid analyzer (Hitachi L-8900, Japan) according to AOAC (1995). The sulfur-containing amino acids were detected according to an oxidation hydrolysis method: about 100 mg meat samples were dissolved in hydrogen peroxide with formic acid (1:9) for 16 h and then added with 0.5 mL sodium bisulfate and 8 mL 6.8 mol/L of HCl, after hydrolysis for 24 h at 110°C, the solution was added with 0.75 mL sodium citrate and 0.15 mL sodium hydroxide before filtered by a 0.45-μm filter. And then sulfur-containing amino acids in the solution was determined by the amino acid analyzer. Tryptophan was determined according to an alkaline hydrolysis method: about 100 mg muscle samples were dissolved in 4 mol/L of lithium hydroxide and then hydrolyzed for 20 h at 110°C. Thereafter, the solution was adjusted for pH by adding HCl and filtered through a 0.45-μm filter. Then the solution loaded on the amino acid analyzer.

#### Fatty acids analysis

Total lipids of meat were extracted using a mixture of polar and non-polar solvents, and then fatty acids were methylated into fatty acid methyl esters (FAME) [16]. The fatty acid profile was determined by gas chromatography (Shimadzu GC-2010, Tokyo, Japan) equipped with flame ionization detector and split injection,100 m length, 0.25 mm i.d., 0.2 μm thickness capillary gas chromatography column (SP-2560; Supelco, Bellefonte, PA, USA). The initial oven temperature was 150°C, which was held for 5 min, then increased to 200°C at a rate of 2°C/min and held for 10 min, then increased to 220°C at 5°C/min and held for 35 min. Helium was used as carrier gas at a flow rate of 1 mL/min. The injector was set at 260°C, and the detector was set at 280°C. FAMEs were identified by compared with the retention times of the standards. The standards were FAME MixC4-C24 (Sigma 18919, Deisenhofen, Germany).

### Statistical analysis

The data on growth performance, dry matter intake (DMI), feed conversion rate, blood biochemical parameters, meat quality traits, amino acids and fatty acids composition were compared using PROC MIXED of SAS (version 9.2, SAS Institute Inc., Gary, NC, USA) with polynomial contrasts to test the effects of mixing ratio for dietary *B. papyrifera* silage, a randomized complete block design was used with diet as fixed effects and beef cattle within diet as a random effect. The data were expressed as mean and accompanied by standard error of the mean. Differences were considered significant at p<0.05.

## RESULTS

### Performance

Beef cattle in 15% group had higher final BW than those in other groups, and beef cattle in 5%, 10%, or 15% group had higher average daily gain (ADG), DMI, and feed conversion rate (FCR) than that in control group (p<0.05) (Table 3).

### Blood metabolites, immune and antioxidant functions

Blood biochemical indexes of beef, TP, ALB, GLB, NEFA, BUN, TC, TG, GLU, HDL-C, and LDL-C, and blood immune function parameters, Ig A, Ig G, Ig M, and TNFα, and anti-oxidation indexes, AOPP, GSH-Px, and CAT, were not affected by the diets supplemented with *B. papyrifera* silage (p>0.1).

The beef cattle in 10% group and 15% group had signifi cantly higher TAC in blood serum than that in control group or 5% group, but blood TAC in 5% group had no significant difference compared with that in control group. Blood SOD in 15% was significantly higher than that in control group, 5% group or 10% group, but no significant difference was observed among control group, 5% group and 10% group. The beef cattle in 15% group had higher blood 8-OHdG than that in control group, 5% group or 10% group, but no significant difference was observed among control group, 5% group, and 10% group. Blood MDA was significantly lower in 15% group than in control group or 5% group, but no significant difference among control group, 5% group and 10% group (Table 4).

### Meat quality traits

Diets supplemented with *B. papyrifera* silage had no effect on dressing rate of beef cattle compared with that in the control group (p>0.05). Meat pH was lower in 15% group than that in control group, 5% group or 10% group, but there was no significant difference in meat pH between control group and 5% group or 10% group (p<0.05). Beef cattle had significantly higher CIE L* in 5% group, 10% group, and 15% group than that in the control group (p<0.05). CIE a* was higher in 10% group than that in the control group or 15% group (p< 0.05), but there is no difference in CIE a* between 5% group and 10% group. The diet with 15% of *B. papyrifera* silage significantly decreased the drip loss of meat compared with the control group, but there was no significant difference among 5% group, 10% group, and 15% group. *B. papyrifera* silage had no significant effect on CIE b* of meat, cooking loss and shearing force of meat (Table 5).

### Amino acids and fatty acids composition in meat

No significantly differences were observed in amino acids composition between groups (p>0.05) (Table 6).

C12:0 was significantly lower in 10% group and 15% group than that in control group (p<0.05), but there was no significant difference between 5% group and control group. C18:2n6c was significantly higher in 15% group than that in control group or 5% group (p<0.05), but no significant difference among control group, 5% group and 10% group. C18:3n3 was significantly higher in 5% group, 10% group, and 15% group than that in control group (p<0.05). C22:3n6 was significantly higher in 15% group than that in control group, 5% group, or 10% group (p<0.05), but no difference was observed among control group, 5% group and 10% group. C22:6n3 was significantly higher in 5% group, 10% group, or 15% group than that in control group, and C22:6n3 was significantly higher in 15% group than that in 5% group or 10% group (p<0.05). The PUFA was significantly higher in 10% group and 15% group than that in control group, but no significant difference between control group and 5% group (Table 7).

## DISCUSSION

### Performance

Beef cattle had significantly higher DMI in 5% group, 10% group, and 15% group than that in control group (p<0.05). It is reported that catalytic foliage supplementation to low-quality roughages may enhance nutrient utilization in sheep [17], and foliage supplementation increased total feed intake of goats [18] with which our result of DMI was consistent. However, our results were different from the results that *B. papyrifera* silage decreased the DMI in dairy cattle [5], which may have been due to the replacement of low quality forage with high quality forage in diets for beef cattle, not like the low quality forage used in the diets for dairy cattle decreasing the feed intake, high quality forage could increase the DMI in beef cattle.

Beef cattle had higher ADG and FCR in all treatment groups than that in control group, which was consistent with the result reported by Patra that foliage supplementation resulted in increased ADG [18], as it is likely due to increased intakes of digestible nutrients by the ruminants.

### Blood metabolites, immune and antioxidant parameters

Blood metabolites and immune parameters reflect health status of animals and the utilization of nutrients. No significant differences were found in the blood TP, ALB, GLB, NEFA, BUN, TC, TG, GLU, HDL-C, LDL-C, IgA, IgG, IgM, and TNFα among groups (p>0.05), and all parameters in the normal range, which indicated that the beef cattle were in healthy status and the metabolic status had no changes due to intake of different amount of *B. papyrifera* silage supplemented in the diets.

Oxidative stress is an imbalance between free radical gen eration and antioxidant system, which is a main contributing factor inducing various disease in animals [19]. It is reported that there are flavonoids with antioxidant function naturally occurring in bark, fruit and leaves of *B. papyrifera* [20,21]. The nutrients with antioxidants could be used to control oxidative stress [22]. SOD is the first line of antioxidant defense that catalyzes the conversion of superoxide radical to hydrogen peroxide [23]. The blood SOD was significantly higher in 15% group than that in control group, 5% group or 10% group (p<0.05), but no statistically significant difference of blood SOD was found among control group, 5% group, and 10% group, which suggested that the diet supplemented with 15% *B. papyrifera* could increase the antioxidative capacity. TAC represents the antioxidant response against the free radicals. The blood TAC was significantly higher in 10% group and 15% group than that in control group or 5% group, which suggested that higher *B. papyrifera* silage in the diet increased blood TAC in beef cattle. MDA is one of the final products of PUFAs peroxidation and accumulates as free radicals increase. In the present study, blood MDA was significantly lower in 15% group than that in control group, 5% group or 10% group, which is consistent with the results that an increase in TAC is not parallel with an increase in MDA in cows [24]. It suggested that some antioxidants in the *B. papyrifera* silage could reduce the oxidation products or oxidant reaction in animal blood, which might decrease oxidative stress. Blood 8-OHdG was significantly lower in 15% group than that in control group, 5% group, or 10% group (p<0.05). 8-OHdG is the most frequently detected analyte representing DNA lesion resulting from oxidative stress and is a biomarker of the oxidative DNA damage and repair. As *B. papyrifera* silage increased in the diet, beef cattle had lower blood 8-OHdG, which suggested that diets with 15% *B. papyrifera* silage could significantly reduce DNA lesions caused by oxidative stress. The *B. papyrifera* silage might have effect on reducing oxidative stress of the beef cattle, and it could be a potential feed stuff with antioxidant capacities for beef cattle.

If the free radicals exceed the antioxidant capacity of the body system, oxidative stress will occur, which will affect the animals’ growth and development in negative way [19]. It is reported that the plant pigments could improve growth performance of livestock through immune status [25] or antioxidant status [26]. In the present study, we found that 15% dietary *B. papyrifera* silage could affect the antioxidant system by increasing blood SOD and TAC, and decreasing MDA, and meanwhile, 15% dietary *B. papyrifera* silage improved the performance by increasing final BW, ADG, DMI, and FCR of beef cattle in 15% group. Higher activities of SOD and TAC enzymes could reduce the oxygen radicals in the system and alleviate the oxidative damage to organisms, and lower MDA indicates less oxidative stress in animals, in which way the growth performance, such as final BW, ADG, DMI, and FCR, were improved in the present study.

### Meat quality traits

Silage produces more lactic acid during fermentation, which might result in lower meat pH, In the present study, meat pH was lower in 15% group that those in other groups, which might be induced by dietary *B. papyrifera* silage in 15% group. Meat color is an important parameter of meat quality and the first impression that the consumers have of meat. Beef cattle had higher CIE L* in 5% group, 10% group, and 15% group than that in control group, and beef cattle had higher CIE a* in 10% group than that in control group, 5% group, or 15% group. It is reported that antioxidant treatments significantly had higher redness (a*) values [27]. *B. papyrifera* contains antioxidant activities as mentioned above. It may have induced a better color value in the present study. Dark meat has a low acid content, i.e., high pH [28], which is consistent with our results that beef cattle fed on the diets with *B. papyrifera* silage had lower pH (p<0.05) but higher CIE L* and a*. Value for redness (a*) below 9.5 are considered dark and not appreciated by consumers [29], and in the present study, beef cattle fed on the diets with *B. papyrifera* had a* value more than 9.5. Drip loss of meat was significantly lower in 15% group than that in control group, 5% group, or 10% group. Lower drip loss indicates a higher quality meat. It is reported that dietary antioxidants could decrease meat drip loss [30] resulting from decreased membrane phospholipase activity [31] by protecting membranes from the action of phospholipases, which could cause a less substantial reduction in the fluidity and finally reduce the drip loss. In the present study, 15% *B. papyrifera* silage supplement in the diet could have the same function in improving meat quality.

### Amino acids and fatty acids composition in meat

The profile of amino acids had no significant difference among groups. The active ingredients of *B. papyrifera* silage, such as polyphenols and flavonoids, might affect the ruminant microorganism on the fatty acid biohydrogenation but not amino acids metabolism.

Ruminant milk and meat might contain more SFA because of the biohydrogenation occurring in the rumen. There are considerable studies focusing on reducing the biohydrogenation by adding more unsaturated fatty acids of feedstuff [32,33], protecting unsaturated fatty acids [34] or supplying feedstuff against biohydrogenation [35] in ruminants’ diets. In the present study, PUFA were significantly higher in 10% and 15% groups than that in control group or 5% group (p<0.05), and it might due to the antimicrobial activities in the diet supplemented with *B. papyrifera* silage, as it is reported that prenylated flavonoids isolated from *B. papyrifera* have antimicrobial activity [36,37] which might affect the rumen microorganism by reducing the biohydrogenation of fatty acids in the rumen, and also dietary antioxidant activity might increase PUFA content in meat [38,39]. We found the similar result in milk of dairy cattle [5]. C22:6n3 (docosahexaenoic acid, DHA) of meat is higher in 5% group, 10% group and 15% group than that in control group (p<0.05). Beef cattle fed on the diet supplemented with *B. papyrifera* silage had higher DHA in meat, which would be beneficial for the consumers’ health.

## CONCLUSION

*B. papyrifera* silage could be used as a new feed resource in finishing beef cattle. Beef cattle fed on diet supplemented with 15% *B. papyrifera* silage could increase their performance by increasing final BW, ADG, DMI, and FCR, enhance the antioxidant functions by decreasing blood 8-OHdG and MDA and increasing blood SOD and TAC, improve the meat quality by lowing pH and drip loss and increasing CIE L*, increase the PUFA and DHA concentration in meat. Polyphenols and flavonoids might be the main constituents responsible for the antioxidant activity and anti-biohydrogenation in the *B. papyrifera* silage. Fatty acids in the diets are biohydrogenated by bacteria in the rumen, and we will focus further study on the effect of *B. papyrifera* silage on the rumen fermentation and bacteria composition and abundance.

## Figures and Tables

**Table 1:** Ingredients and nutrition levels of diets (air dry basis, %)

Items	Control group	5% group	10% group	15% group	*B. papyrifera* silage
Ingredients					
Flaked corn	27.0	27.0	27.0	27.0	-
Maize meal	14.5	15.5	17.0	17.8	-
Extruded soybean	2.90	2.50	2.50	2.50	-
Wheat bran	7.72	8.00	8.00	6.85	-
Rice bran	26.1	25.8	26.0	26.4	-
Wheat middling	2.4	1.7	0.0	0.0	-
NaCl	0.33	0.33	0.33	0.33	-
NaHCO_3_	0.72	0.72	0.72	0.72	-
Premix1)	1.0	1.0	1.0	1.0	-
Corn straw ammoniation	17.3	12.5	7.5	2.5	-
*B. papyrifera* silage	0.0	5.0	10.0	15.0	-
Total	100	100	100	100	-
Nutrient levels					
DM (%)	54.2	55.7	56.0	57.3	21.2
CP (% DM)	9.83	9.99	10.35	10.71	16.0
EE (% DM)	3.43	3.40	3.51	3.58	3.34
NDF (% DM)	45.2	43.6	42.0	40.3	45.5
ADF (% DM)	24.9	24.1	23.4	22.8	29.0
Ash (% DM)	6.78	6.94	7.12	7.26	11.6
Water soluble carbohydrate (% DM)	-	-	-	-	4.18
Lactic acid (g/kg DM)	-	-	-	-	87.3
Acetic acid (g/kg DM)	-	-	-	-	20.9
Propionic acid (g/kg DM)	-	-	-	-	1.1
Lactic acid/total acid	-	-	-	-	79.75
Acetic acid/total acid	-	-	-	-	19.23
Propionic acid/total acid	-	-	-	-	1.02
Ammonia-N/total N	-	-	-	-	10.45

*B. papyrifera*, *Broussonetia papyrifera*; DM, dry matter; CP, crude protein; EE, ether extract; NDF, neutral detergent fiber; ADF, acid detergent fiber.

1) One kilogram of premix contains 600,000 IU vitamin A, 100,000 IU vitamin D, 4,000 IU vitamin E, 3,000 mg Fe, 2,000 mg Cu, 2,500 mg Mn, 8,000 mg Zn, 60 mg Se, 100 mg I, 20 mg Co.

**Table 2:** Fatty acids composition of the diets

% of total fatty acids	Control group	5% group	10% group	15% group
C8:0	0.01	0.01	0.01	0.02
C10:0	0.02	0.03	0.02	0.03
C12:0	0.09	0.08	0.07	0.08
C14:0	1.45	1.47	1.42	1.43
C15:0	0.03	0.02	0.03	0.03
C16:0	17.6	16.4	15.9	16.3
C17:0	0.12	0.17	0.15	0.11
C18:0	2.95	3.14	4.04	3.83
C18:1n9c	23.7	24.2	25.3	24.9
C18:2n6c	51.4	51.9	50.9	50.7
C18:3n3	2.27	2.14	2.42	2.09
C20:0	0.42	0.45	0.35	0.56
C22:0	0.14	0.13	0.09	0.11
C24:0	0.03	0.05	0.03	0.04

**Table 3:** Effect of *Broussonetia papyrifera* silage on performance of black Angus

Items	Control group	5% group	10% group	15% group	SEM	p-value
Initial BW (kg)	494	494	495	496	17.4	0.91
Final BW (kg)	585^b^	596^ab^	608^ab^	616^a^	6.7	0.05
ADG (kg/d)	0.91^b^	1.10^a^	1.13^a^	1.18^a^	0.013	0.03
DMI (kg/d)	7.16^b^	8.23^a^	8.44^a^	8.45^a^	0.11	0.04
FCR	0.12^c^	0.13^b^	0.14^a^	0.14^a^	0.001	0.04

SEM, standard error of the mean; BW, body weight; ADG, average daily gain; DMI, dry matter intake; FCR, feed conversion rate.

a–c Means within row with different superscripts differ significantly (p<0.05).

**Table 4:** Effect of *Broussonetia papyrifera* silage levels on blood biochemical parameters, immune indexes and anti-oxidant indexes of black Angus

Items	Control group	5% group	10% group	15% group	SEM	p-value
Biochemical indexes						
TP (g/L)	75.4	76.3	75.3	79.5	3.06	0.96
ALB (g/L)	24.5	22.1	24.3	25.6	0.87	0.57
GLB (g/L)	50.9	54.2	51.0	53.9	2.49	0.96
NEFA (μmol/L)	482	496	538	514	12.7	0.49
BUN (mmol/L)	3.88	3.58	3.63	4.05	0.15	0.67
TC (mmol/L)	3.30	3.39	3.40	3.14	0.16	0.94
TG (mmol/L)	0.11	0.12	0.15	0.13	0.011	0.76
GLU (mmol/L)	5.43	4.30	5.30	5.60	0.31	0.48
HDL-C (mmol/L)	2.28	2.40	2.33	2.19	0.077	0.42
LDL-C (mmol/L)	0.71	0.73	0.81	0.69	0.044	0.84
Immune indexes						
IgA (g/L)	0.79	0.93	0.89	0.83	0.064	0.91
IgG (g/L)	8.36	9.24	9.46	7.79	0.29	0.12
IgM (g/L)	0.61	0.71	0.74	0.64	0.046	0.78
TNFα (ng/mL)	1.46	1.50	1.45	1.32	0.045	0.57
Anti-oxidation indexes						
SOD (U/mL)	131^b^	131^b^	134^b^	151^a^	3.22	0.043
TAC (U/mL)	11.8^b^	12.3^b^	14.2^a^	15.7^a^	0.51	0.002
8-OHdG (ng/mL)	0.57^a^	0.51^a^	0.48^ab^	0.33^b^	0.020	0.024
AOPP (μmol/L)	19.7	19.4	19.4	18.1	0.40	0.48
MDA (μmol/L)	3.84^a^	3.66^a^	3.21^ab^	2.62^b^	0.18	0.033
GSH-Px (U/mL)	849	833	842	837	10.3	0.96
CAT (U/mL)	5.93	4.00	5.10	4.52	0.27	0.062

SEM, standard error of the mean; TP, total protein; ALB, albumin; GLB, globulin; NEFA, non-esterified fatty acid; BUN, blood urea nitrogen; TC, total cholesterol; TG, total glyceride; GLU, glucose; HDL-C, high density lipoprotein cholesterol; LDL-C, low density lipoprotein cholesterol; Ig, immunoglobulin; TNFα, tumor necrosis factor α; SOD, superoxide dismutase; TAC, total antioxidant capacity; 8-OHdG, 8-hydroxydeoxyguanosine; AOPP, advanced oxidation protein products; MDA, malondialdehyde; GSH-Px, glutathione peroxidase; CAT, catalase.

a,b Means within row with different superscripts differ significantly (p<0.05).

**Table 5:** Effect of *Broussonetia papyrifera* silage levels on beef quality of black Angus

Items	Control group	5% group	10% group	15% group	SEM	p-value
Dressing rate (%)	57.4	56.5	57.2	56.66	0.32	0.83
pH	6.54^a^	6.69^a^	6.50^a^	6.16^b^	0.056	<0.0001
CIE L*	25.0^b^	27.4^a^	27.7^a^	28.9^a^	0.44	0.004
CIE a*	8.57^b^	12.05^ab^	15.10^a^	9.72^b^	0.83	0.009
CIE b*	4.72	4.53	4.64	4.92	0.16	0.88
Cooking loss (%)	29.8	30.5	28.5	28.7	0.54	0.22
Drip loss (%)	19.6^a^	16.9^ab^	16.1^ab^	13.3^b^	0.82	0.029
Shearing force (N)	32.7	35.5	33.3	33.4	0.85	0.71

SEM, standard error of the mean; CIE, Commission International DeI’Eclairage.

a,b Means within row with different superscripts differ significantly (p<0.05).

**Table 6:** Effect of *Broussonetia papyrifera* silage levels on amino acids composition of meat in black Angus

Amino acids (% of total amino acids)	Control group	5% group	10% group	15% group	SEM	p-value
Aspartic acid	6.65	6.89	6.49	6.50	0.14	0.77
Threonine	3.26	3.41	3.20	3.19	0.069	0.70
Serine	2.79	2.89	2.72	2.73	0.057	0.75
Glutamic acid	10.64	11.13	10.46	10.36	0.23	0.70
Proline	2.69	2.74	2.59	2.58	0.059	0.76
Glycine	3.15	3.16	3.06	3.04	0.064	0.89
Alanine	2.92	2.98	2.81	2.83	0.064	0.80
Valine	3.56	3.75	3.49	3.49	0.081	0.69
Isoleucine	3.50	3.62	3.37	3.39	0.081	0.73
Leucine	5.99	6.07	5.70	5.84	0.13	0.81
Tyrosine	2.41	2.52	2.38	2.31	0.061	0.72
Phenylalanine	2.90	3.03	2.85	2.85	0.064	0.76
Histidine	2.91	3.04	2.78	2.87	0.070	0.66
Lysine	6.58	6.86	6.41	6.45	0.14	0.74
Arginine	4.77	4.74	4.42	4.58	0.107	0.70
Cystine	0.85	0.88	0.81	0.83	0.018	0.57
Methionine	2.18	2.28	2.06	2.15	0.047	0.47
Tryptophan	0.83	0.89	0.84	0.84	0.017	0.62

SEM, standard error of the mean.

**Table 7:** Effect of *Broussonetia papyrifera* silage levels on fatty acids composition in meat of black Angus

Fatty acids (% total fatty acids)	Control group	5% group	10% group	15% group	SEM	p-value
C8:0	0.03	0.05	0.04	0.03	0.003	0.16
C10:0	0.06	0.05	0.04	0.04	0.002	0.19
C12:0	0.07^a^	0.06^ab^	0.05^b^	0.05^b^	0.003	0.018
C14:0	2.85	2.52	2.26	2.29	0.099	0.13
C14:1	0.64	0.50	0.48	0.50	0.045	0.65
C15:0	0.35	0.35	0.33	0.33	0.010	0.84
C16:0	28.9	27.4	27.1	27.8	0.31	0.26
C16:1	2.98	2.44	2.42	2.67	0.42	0.13
C17:0	0.81	0.90	0.89	0.87	0.018	0.37
C18:0	17.2	18.5	17.3	17.0	0.67	0.66
C18:1n9c	41.1	41.5	42.8	42.0	0.72	0.27
C18:2n6c	3.44^b^	3.65^b^	4.15^ab^	4.47^a^	0.13	0.017
C18:3n3	0.24^b^	0.30^a^	0.30^a^	0.31^a^	0.008	0.016
C20:0	0.17	0.17	0.16	0.15	0.005	0.75
C20:1	0.17	0.16	0.18	0.16	0.013	0.74
C21:0	0.07	0.07	0.08	0.07	0.004	0.88
C20:2	0.01	0.02	0.02	0.01	0.001	0.27
C20:3n3	0.01	0.01	0.02	0.01	0.001	0.31
C20:3n6	0.13^b^	0.17^ab^	0.18^ab^	0.23^a^	0.013	0.018
C20:4n6	0.52	0.80	0.78	0.63	0.070	0.48
C20:5n3	0.02	0.03	0.03	0.03	0.004	0.72
C22:0	0.05	0.05	0.05	0.05	0.003	0.72
C22:1n9	0.02	0.04	0.04	0.03	0.005	0.34
C22:6n3	0.01^c^	0.02^b^	0.02^b^	0.03^a^	0.002	0.0015
C24:0	0.15	0.20	0.19	0.17	0.015	0.72
C24:1	0.04	0.05	0.04	0.04	0.003	0.97
SFA	50.6	50.4	48.5	48.9	0.73	0.86
UFA	49.4	49.6	51.5	51.1	0.73	0.86
MUFA	45.0	44.6	46.0	45.4	0.88	0.68
PUFA	4.39^b^	5.01^ab^	5.51^a^	5.71^a^	0.19	0.041
n-6	5.46	5.13	4.40	4.29	0.24	0.28
n-3	0.37	0.37	0.34	0.38	0.22	0.91
n-6/n-31)	14.6	13.6	13.0	12.4	0.42	0.60

SEM, standard error of the mean; SFA, saturated fatty acid; UFA, unsaturated fatty acid; MUFA, monounsaturated fatty acid; PUFA, polyunstaturated fatty acid.

1) It is the ratio of n-3 to n-6, not the percentage of total fatty acids.

a–c Means within row with different superscripts differ significantly (p<0.05).
